# Molecular Origins of the Compatibility between Glycosaminoglycans and Aβ40 Amyloid Fibrils

**DOI:** 10.1016/j.jmb.2017.07.003

**Published:** 2017-08-04

**Authors:** Katie L. Stewart, Eleri Hughes, Edwin A. Yates, David A. Middleton, Sheena E. Radford

**Affiliations:** 1 Astbury Centre for Structural Molecular Biology, School of Molecular and Cellular Biology, University of Leeds, Leeds LS2 9JT, UK; 2 Department of Chemistry, University of Lancaster, Lancaster LA1 4YB, UK; 3 Department of Biochemistry, Institute of Integrative Biology, University of Liverpool, Liverpool, L69 7ZB, UK

**Keywords:** AD, Alzheimer's disease, GAGs, glycosaminoglycans, Aβ40, A-beta 40-residue peptide, Aβ42, A-beta 42-residue peptide, SSNMR, solid-state NMR, LMWH, low-molecular-weight heparin, HS, heparan sulfate, WT, wild-type, NS, *N*-sulfate, 6-OS, 6-*O*-sulfate, 2-OS, 2-*O*-sulfate, amyloid β, glycosaminoglycans, Alzheimer's disease, amyloid fibrils, heparin binding

## Abstract

The Aβ peptide forms extracellular plaques associated with Alzheimer's disease. In addition to protein fibrils, amyloid plaques also contain non-proteinaceous components, including glycosaminoglycans (GAGs). We have shown previously that the GAG low-molecular-weight heparin (LMWH) binds to Aβ40 fibrils with a three-fold-symmetric (3Q) morphology with higher affinity than Aβ40 fibrils in alternative structures, Aβ42 fibrils, or amyloid fibrils formed from other sequences. Solid-state NMR analysis of the GAG–3Q fibril complex revealed an interaction site at the corners of the 3Q fibril structure, but the origin of the binding specificity remained obscure. Here, using a library of short heparin polysaccharides modified at specific sites, we show that the *N*-sulfate or 6-*O*-sulfate of glucosamine, but not the 2-*O*-sulfate of iduronate within heparin is required for 3Q binding, indicating selectivity in the interactions of the GAG with the fibril that extends beyond general electrostatic complementarity. By creating 3Q fibrils containing point substitutions in the amino acid sequence, we also show that charged residues at the fibril three-fold apices provide the majority of the binding free energy, while charged residues elsewhere are less critical for binding. The results indicate, therefore, that LMWH binding to 3Q fibrils requires a precise molecular complementarity of the sulfate moieties on the GAG and charged residues displayed on the fibril surface. Differences in GAG binding to fibrils with distinct sequence and/or structure may thus contribute to the diverse etiology and progression of amyloid diseases.

## Highlights

• Through substitutions in LMWH and Aβ40, details of fibril–GAG binding are revealed.
• Both charge and topology contribute to binding of heparin to Aβ40 3Q fibrils.
• The 6-*O*-sulfate, but not the 2-*O*-sulfate, of LMWH is required for fibril binding.
• Charged residues at the fibril apices dominate GAG binding free energy.
• Rules rationalizing binding of GAGs to amyloid are presented.

## Graphical Abstract

**Figure:**
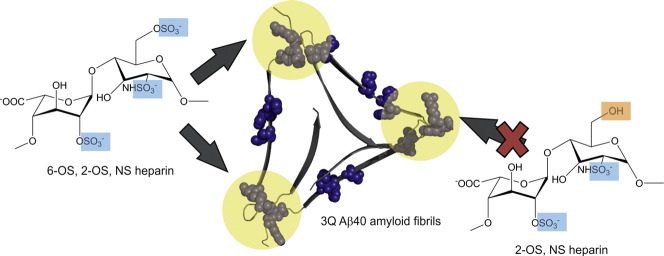

## Introduction

Aggregation of proteins and peptides into amyloid fibrils is responsible for more than 50 human diseases [1]. One of the most well-documented amyloid pathologies is Alzheimer's disease (AD), which results from the extracellular deposition of fibrils formed from the Aβ peptide, which ranges from 38 to 43 amino acids in length, along with intracellular deposits of the protein tau [2], [3]. Amyloid plaques also contain an assortment of accessory molecules including nucleic acids [4], lipids [5], metal ions [6], and glycosaminoglycans (GAGs) [7]. GAGs are linear sulfated polysaccharides, which include heparan sulfate (HS), the structure of which varies between mammals and between different tissues [8], including the brain [9], and its less ubiquitous, but more homogeneous and highly sulfated variant heparin. Heparin and its shorter derivative, low-molecular-weight heparin (LMWH), have been shown to interact with a variety of amyloid proteins *in vitro* including amylin [10], [11], α-synuclein [12], transthyretin [13], [14], β_2_-microglobulin [15], [16], gelsolin [17], [18], tau [19], [20], and Aβ40/42 [21], [22]. There is also increasing evidence that heparin and other GAGs may be active participants in the formation of amyloid fibrils. Heparin has been shown to accelerate fibril formation [21], [23], enhance fibril stability [24], [25], and decrease amyloid toxicity [26], [27]. As a consequence, prevention of amyloid–GAG interactions has been considered as an anti-amyloid strategy [28], [29], [30]. In addition to heparin and HS, a number of other natural GAGs have been identified in extracellular amyloid plaques, including chondroitin sulfate [31], [32], [33], dermatan sulfate [34], and keratan sulfate [35], all of which, like heparin and HS, comprise sulfated disaccharide units. These sulfate groups are proposed as a requirement for molecular recognition *via* electrostatic interactions between the GAG and basic amino acid side chains of binding partners [36]. However, few models of an amyloid–GAG structure have been determined to date [37], [38], leaving unresolved the question of how GAGs recognize the cross-β structure of amyloid. Studies of soluble (non-amyloid) proteins bound to GAGs suggest that there is a complex relationship between protein binding and charge disposition, conformation, and flexibility of the polysaccharide [39]. Indeed, a recent survey of the primary sequences of 437 heparin binding proteins confirmed that the notion of consensus binding sequences can be disregarded [40]. Instead, the contacts between GAGs and proteins typically comprise short, well-spaced positive (lysine or arginine) or potentially positive (histidine) amino acids and those capable of hydrogen bonding (glutamine, asparagine) alternating with hydrophobic residues. Similar complexity in relation to amyloid fibrils may also be expected to occur, especially given the array of different amyloid structures that have recently been determined using solid-state NMR (SSNMR) [41], [42], [43], [44]. However, a systematic study of the biochemical origins of GAG–amyloid binding has yet to be reported. Here, we characterize LMWH–amyloid interactions in molecular detail, employing a unique (single) structure of Aβ40 fibrils determined by Paravastu and colleagues [45], known as 3Q. Using substitutions in LMWH and in the protein sequence, we reveal a surprising specificity in binding, which is dependent on the substitution pattern of the GAG and the display of positive amino acid side chains in the fibril three-dimensional structure.

Despite all amyloid fibrils sharing a cross-β architecture [2], [46], amyloid structures can be highly varied, with differences in the number, orientation, and organization of the β-strands, even for fibrils formed from the same sequence [2], [41], [47], [48]. In previous work, we showed that differences in amyloid sequence and structure can have a profound effect on LMWH binding [37], [49]. For example, LMWH binds with different affinity to fibrils of Aβ40 with distinct morphologies (3Q, 2A, and fibrils with mixed morphology formed from Aβ40 *de novo*; Fig. S1 and [37]), while little binding is observed under the same conditions for fibrils of Aβ42, Aβ16–22, and amylin (Fig. S1 and [37]). Mapping the binding sites of 3Q fibrils for LMWH using SSNMR revealed specific chemical shift perturbations for residues, which lie at the apices of the 3Q fibril structure, indicating that the fibril architecture itself is important in dictating binding [37]. These findings suggest that GAG–amyloid interactions may be more specific than perceived hitherto, in which binding was thought to be dominated by non-specific electrostatic complementarity of the negatively charged GAG and positively charged fibril surface or a simple linear consensus sequence [50].

Given the common finding that LMWH co-localizes with amyloid fibrils, we sought here to unravel the determinants of the binding site of LMWH to Aβ40 fibrils of 3Q morphology by altering systematically both the GAG and protein chemistries. The interactions between an array of short heparin molecules containing specific sulfate substitutions and Aβ40 sequence variants assembled by seeding into a 3Q fibril morphology are characterized in detail using *in vitro* binding assays, complemented by SSNMR. The results reveal the importance of both the GAG substituents and peptide sequence in determining the specific interaction site of LMWH for 3Q fibrils and rationalize why amyloid fibril–GAG interactions are commonly observed *in vitro* and *in vivo* [51], [52], [53].

## Results

### Forming Aβ40 3Q fibrils by seeded growth

**Fig. 1:**
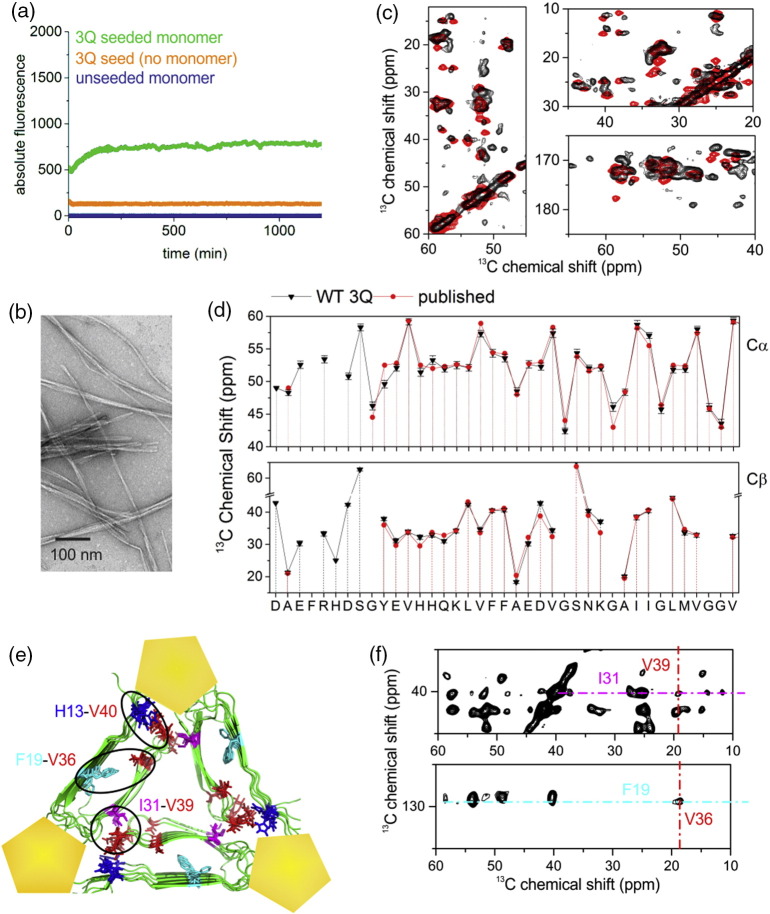
Aβ40 fibrils of 3Q morphology are produced by seeded growth. (a) Seeded growth 5% (v/v) of 3Q fibril seeds with Aβ40 WT monomers (20 μM) monitored by ThT fluorescence. (b) TEM image of fibrils formed in panel A after 24 h. The scale bar represents 200 nm. (c) Three regions of a 2D ^13^C–^13^C SSNMR spectrum (with 50-ms DARR mixing) of Aβ40 fibrils prepared with 3Q seeding. The experimental spectrum (black) is overlaid with a simulated spectrum (red) based on the reported ^13^C chemical shifts for the 3Q morphology [45]. (d) ^13^C Cα and Cβ chemical shifts measured from the spectrum in panel C compared with previous published assignments by Paravastu and colleagues [45]. Error bars on the current data represent ± half the line widths measured at half peak height, with an average of 1.07 ppm across the entire sequence. (e) Structure of the 3Q model of Aβ40 (viewed down the fibril axis) based on SSNMR restraints (PDB ID: 2LMQ[45]), showing the previously determined LMWH binding sites (yellow pentagons). Circled regions highlight three long-range couplings between residues observed in Ref. [44], which are diagnostic of the hairpin structure (H13–V40, F19–V36) and of the quaternary packing arrangement in the 3Q morphology (I31–V39). (f) Regions of a 2D ^13^C–^13^C SSNMR spectrum (200-ms DARR mixing) showing long-range F19–V36 and I31–V39 cross peaks. Spectra are shown in Fig. S2 and assignments in Table S1.

To investigate LMWH–Aβ40 fibril interactions, Aβ40 fibrils with the 3Q morphology were prepared by seeded elongation of wild-type (WT) 3Q fibril seeds (kindly provided by Tycko [45]), with Aβ40 monomers uniformly labeled with ^13^C and ^15^N formed by recombinant expression in *Escherichia coli* (Materials and Methods) [49], [54]. Fibril formation was monitored under quiescent growth conditions using thioflavin T (ThT) fluorescence, and the morphology of the resulting fibrils was assessed using negative stain transmission electron microscopy (TEM). The results showed clear evidence for seeded fibril growth in which an immediate and rapid increase in ThT fluorescence occurs upon the addition of seed under the conditions employed (Fig. 1a). Negative stain TEM revealed linear, unbranched fibrils (Fig. 1b), although the fibril “twist” identified in Ref. [45] was not discernible in our hands. SSNMR was used to verify the formation of 3Q fibrils (Fig. 1c–f) [45], [55]. Two-dimensional (2D) ^13^C–^13^C dipolar-assisted rotational resonance (DARR) spectra showed sharp and well-dispersed resonances, consistent with generation of a specific fibril structure [37], [45], [49] (Figs. 1c and S2). Using these experiments, 95% of the Cα ^13^C resonances could be assigned (Table S1), although ambiguous ^13^C chemical shifts of the isoleucine side chains were observed, as reported previously [55]. The chemical shifts obtained correspond closely to previously published values for fibrils of 3Q morphology [45], [49], [55] (Fig. 1d), consistent with the production of fibrils of the 3Q type, with no evidence for formation of other polymorphs or fibrils formed without seeding, which give rise to broad resonances consistent with sample heterogeneity [49]. Previously, 3Q fibrils were shown to differ from other fibril morphologies through a I31–V39 inter-peptide cross peak, in addition to key intra-peptide cross peaks F19–I32, F19–V36, and H13–V40 that are more generally characteristic of the loop type structure [45] (Fig. 1e). In accord with the 3Q morphology, cross peaks assigned to inter-peptide I31–V39 coupling (Fig. 1e, f, top) and cross peaks assigned to intra-peptide F19–V36 (Fig. 1e, f, bottom), H13–V40, and F19–I32 (Figs. 1e and S2) coupling were visible in our sample of WT 3Q Aβ40 fibrils by SSNMR. These cross peaks were previously identified and assigned in spectra of selectively ^13^C-labeled 3Q fibrils ([45] Fig. 3). These 3Q-seeded Aβ40 fibrils were then used to carry out an analysis of the factors involved in GAG–3Q fibril binding described below.

### Sulfate-specific and disaccharide structure-selective interactions of 3Q fibrils with modified heparins

**Fig. 2:**
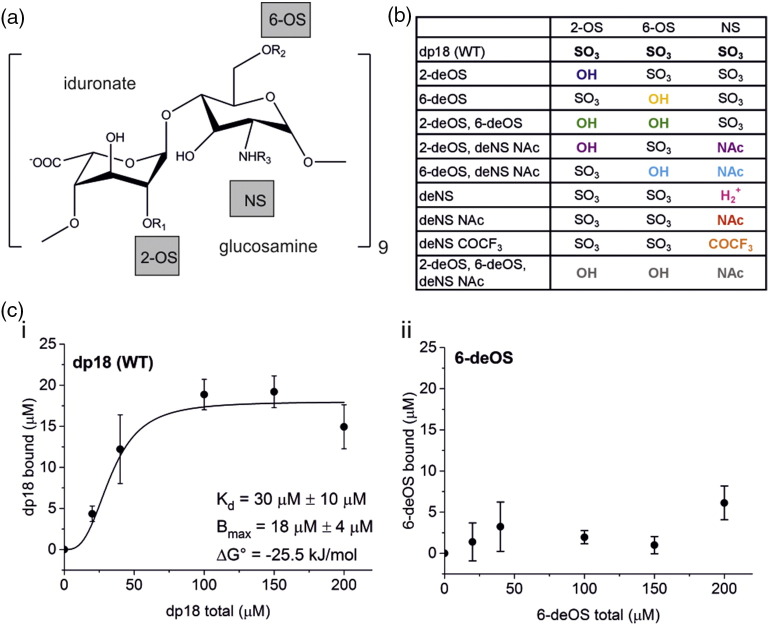
Modifications of heparin substituents affect binding to the 3Q fibril. (a) Structure of a heparin disaccharide unit of dp18 with labeled sulfate groups. (b) List of modifications to the heparin structure based on panel A, with modifications shown in color. (c) Binding curves of modified heparins to 3Q fibrils of Aβ40. Each data point represents the average of three replicates with standard deviation at each heparin concentration. *B*_max_, *K*_d_, and Δ*G*° binding values are reported where binding was detected. The solid line, where applicable, was obtained by non-linear least-squares fitting of a Hill function. Additional heparin variant binding data are shown in Fig. S3 and Table S2.

LMWH binding to 3Q fibrils was first examined from the perspective of the disaccharide units from which heparin is composed (Fig. 2a). Although several studies of GAG binding to amyloid proteins have been performed to date [12], [13], [21], [56], few have investigated the specificity of the GAG structure on the binding affinity to its target protein [37], [38]. Heparin contains alternating α-D-glucosamine and uronic acid substituents, with sulfate modifications commonly at the 2-*O* position on α-L-iduronate and the 2-(amino) and 6-*O* positions on d-glucosamine (Fig. 2a). The removal of sulfate groups from heparin and, in the case of *N*-sulfate (NS), its specific replacement by *N*-acetyl, can be achieved chemically, producing a library of systematically modified heparin polysaccharides with predominantly homogeneous substitution patterns [57]. These modifications enable detailed exploration of the relationship between charge content and structure of the GAG in determining affinity for 3Q fibrils.

Previous work demonstrated that heparin chains above two disaccharide units (dp4) bind 3Q fibrils with similar affinity, with a Δ*G*° binding of ca. 25 kJ/mol [37]. To investigate the role of different heparin substituents on 3Q binding, a series of modified heparin molecules was created using polysaccharides varying in length from 8 to 14 disaccharide units (dp16–dp28) containing sulfate to hydroxide substitutions at the 2-*O*-sulfate (2-OS) and/or 6-*O*-sulfate (6-OS) positions, and/or a variety of substitutions at the 2-NS position (removal of sulfate or substitution with trifluoroacetyl; Fig. 2a–b). (Note that the difference in GAG chain length of the modified GAGs examined here does not affect binding affinity [37].) While the importance of sulfate moieties in amyloid–heparin binding has been noted previously [51], [58], analysis of the relative importance of individual groups in the affinity of this GAG for amyloid has not been reported.

**Fig. 3:**
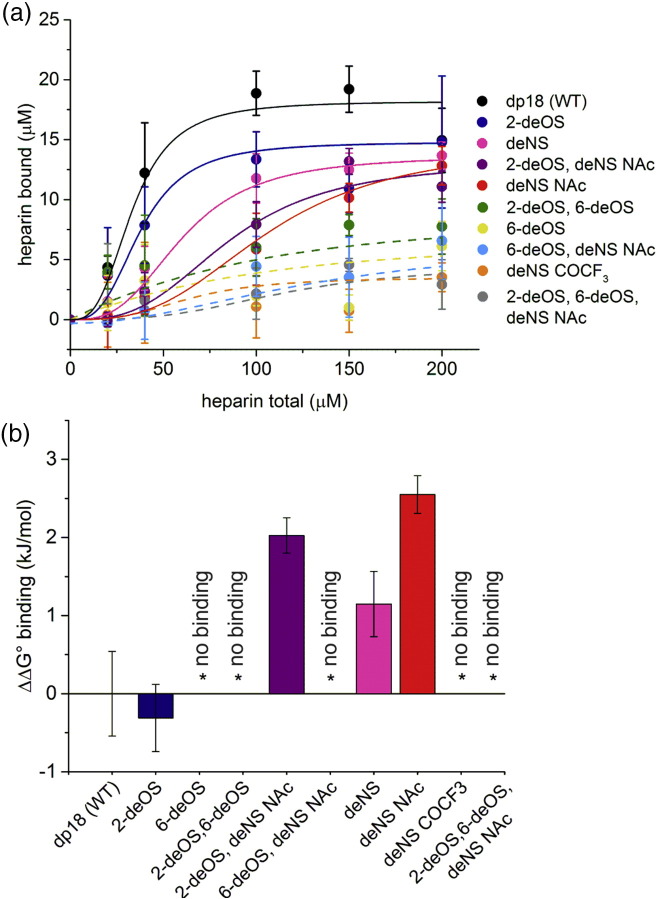
Summary of heparin variants binding to 3Q fibrils of Aβ40. (a) Binding of 3Q fibrils to all modified heparin constructs tested overlaid for comparison. Solid lines indicate heparin variants for which binding could be determined. Dashed lines indicate heparin variants that show little to no binding to 3Q fibrils and for which a *K*_d_ value could not be determined. Lines were obtained by non-linear least-squares fitting of a Hill function. Colors correspond to Fig. 2b. (b) Comparison of ΔΔ*G*° binding of modified heparins to 3Q fibrils, relative to unmodified heparin (dp18), shown in Fig. 2c-i. Asterisks denote heparin variants that showed little or no binding. Error bars depict the standard deviation over three replicate assays.

An *in vitro* assay was employed to measure the binding affinity of the heparin variants to 3Q fibrils [49]. In this assay, the concentration of GAG bound to 3Q fibrils was quantified by pelleting GAG-bound fibrils by centrifugation and determining the saccharide content in the supernatant by enzymatic digestion with heparinase II followed by quantification using UV spectroscopy (Materials and Methods) [49]. Binding curves were produced from three replicate experiments, and the resulting data were fit using the Hill equation to determine an apparent *K*_d_ of binding (Figs. 2c, 3, and S3). This value was compared with analysis of the binding of dp18 (WT) heparin to 3Q fibrils (*K*_d_ = 30 ± 10 μM, Fig. 2c-i) and used to compute the difference in *K*_d_ and hence the ΔΔ*G*° binding for each GAG variant (Fig. 3a–b, Table S2; Materials and Methods). As expected [37], [59], removal of all negative charges (2-deOS, 6-deOS, deNS NAc) resulted in no detectable binding of the modified GAG to 3Q fibrils (Fig. S3A). Similarly, the 6-deOS; 2-deOS, 6-deOS; 6-deOS deNS NAc; and deNS, COCF_3_ heparin variants failed to bind 3Q fibrils or bound too weakly for a *K*_d_ to be determined (Figs. 2c-ii and S3B–D, Table S2). These results mirror previous findings [36] that have shown that electrostatic interactions are important for GAG–amyloid binding. Most interesting, however, was the finding that the sulfate groups at each position differ in their effect on 3Q binding. For example, substitution of the 2-OS with hydroxyl (2-deOS) showed no detectable effect on GAG binding (Fig. S3E), despite the documented effect that this modification has on both the conformation of the iduronate residue and the geometry of the glycosidic linkage [57], [60], [61]. By contrast, removal of the 6-OS (6-deOS) reduced affinity to such an extent that binding could not be measured over the concentration range tested (Fig. 2c-ii). While substitution at the 2-OS iduronate position may alter the orientation of the glycosidic linkage, as well as the equilibrium of boat, chair, and skew-boat conformations in iduronate, substitution of the 6-OS glucosamine position alters the global heparin conformation less dramatically [57], [60], [61]. Hence, the 6-OS, but not the 2-OS, is required for heparin binding to 3Q fibrils.

A more complex picture emerges from substitution of the glucosamine NS. Removal of this moiety (deNS) resulted in a small decrease in binding affinity (*K*_d_ = 54 ± 9 μM; Fig. S3F). Binding is decreased more significantly if the NS is replaced with *N*-acetyl (deNS NAc, *K*_d_ = 95 ± 9 μM; Fig. S3G). Surprisingly, replacement of the NS with *N*-trifluoroacetyl (deNS COCF_3_) eliminated binding (Fig. S3D). Substitution of two sulfates produced an intermediate effect (2-deOS, deNS NAc, *K*_d_ = 77 ± 7 μM; Fig. S3H), highlighting the importance of specific sulfate moieties in the binding affinity to Aβ40 3Q fibrils and possible compensatory effects of the substitutions [62]. Taken together (Fig. 3a–b), the results show that individual sulfates contribute differently to 3Q–LMWH binding, with the 6-OS being critical, the 2-OS playing a minor role, and the NS group showing substitution-specific effects, pointing to the glucosamine residue as particularly important for dictating binding affinity to 3Q fibrils.

### Binding of 3Q fibril mutants to LMWH reveals the role of different residues in GAG binding

**Fig. 4:**
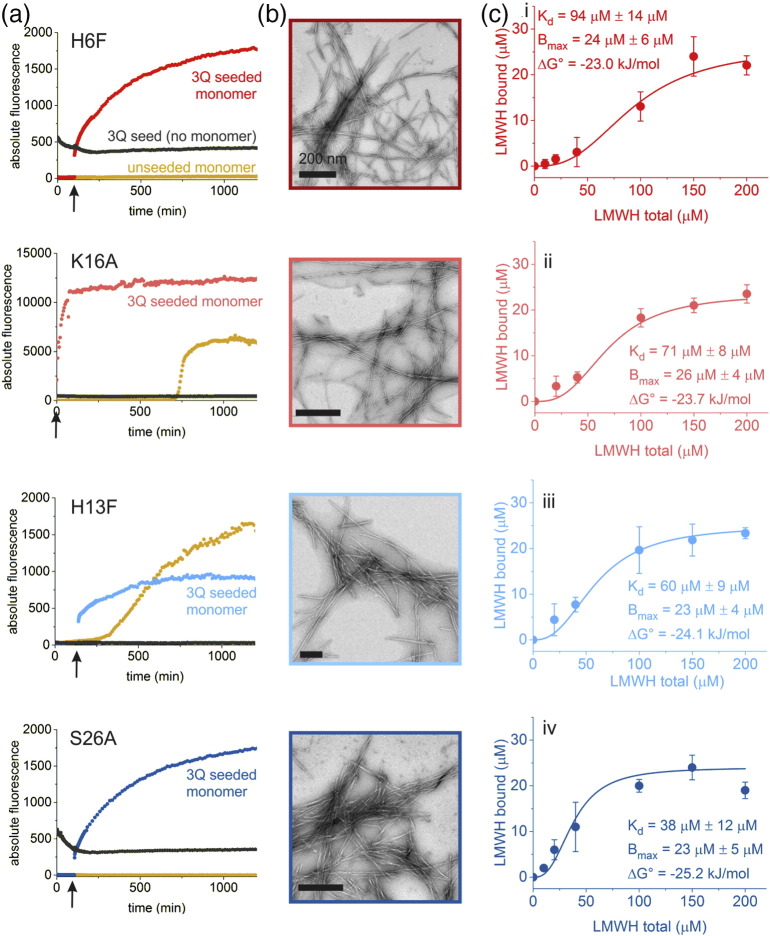
Binding of 3Q fibril variants H6F, K16A, H13F, and S26A to LMWH shows a range of binding free energies. (a) ThT fluorescence confirms that 3Q seeds from WT Aβ40 can be used to seed fibril formation of Aβ40 variants. Seed [5% (v/v)] was added to each monomeric variant at or near the start of incubation, as indicated by an arrow on each panel, causing a rapid increase in fluorescence (red or blue). Each curve shown is a representative based on four replicates. (b) TEM confirms the presence of fibrils after 24 h of seeded growth. (c) Binding curves of variant 3Q fibrils to LMWH indicate a range of binding free energies. Each data point represents the average of three replicates (with standard deviation) at each LMWH concentration. The solid line was obtained by non-linear least-squares fitting of a Hill function. Additional binding data are found in Fig. S7 and Table S4. Colors of the panels correspond to the ΔΔ*G*° binding gradient used in Fig. 5.

**Fig. 5:**
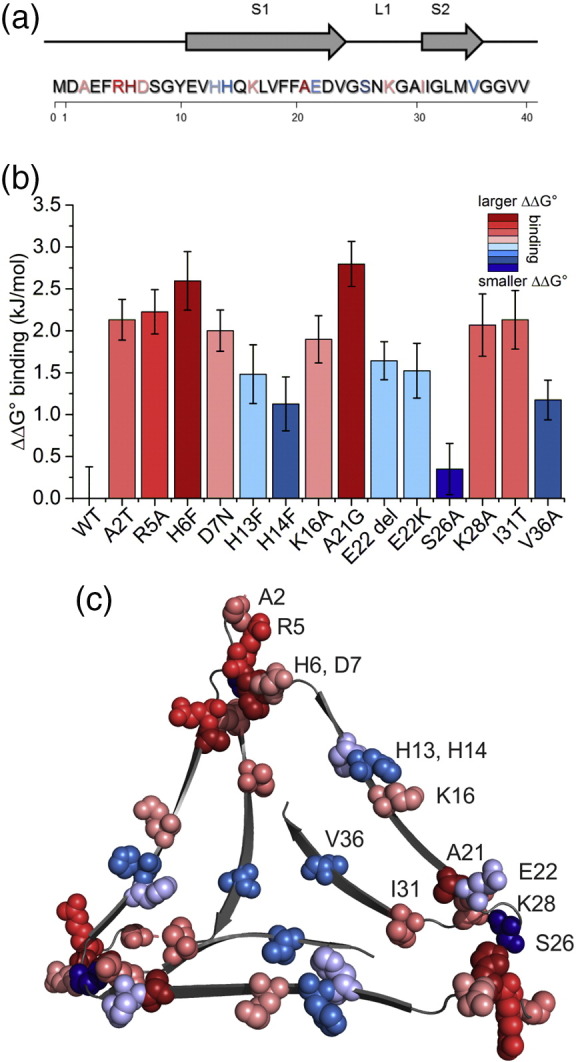
Single-substitution variants highlight the importance of the 3Q fibril “corners” in LMWH binding. (a) The sequence of Aβ40 with the locations of strands and substitution positions in the 3Q morphology shown. (Note that recombinant Aβ40 contains an N-terminal methionine residue at position 0.) (b) Comparison of ΔΔ*G*° values obtained from the binding assay for 3Q variants, relative to WT 3Q fibrils, colored from smaller (blue) to larger (red) changes in ΔΔ*G*° binding. Error bars depict variation in binding between three replicate assays. (c) The Aβ40 3Q fibril structure (from PDBID 2LMQ[45] with an added disordered N-terminus) with substituted residues mapped as space-filling models, colored as in parts A and B.

In order to assess the heparin-3Q fibril interaction from the perspective of different amino acid side chains in the fibril structure, single-residue substitution variants of Aβ40 were produced and assembled into 3Q fibrils by elongation of WT 3Q seeds with each monomer (Figs. 4a and S4). Substitutions were selected in residues which exhibited significant chemical shift differences upon LMWH binding (≥ 0.4 ppm for Cα/β/γ), including R5, H6, H13, H14, E22, S26, and I31; Table S3) [49]. Amino acid substitutions implicated in familial AD (A2T [63], D7N [64], A21G [65], E22Δ [66], and E22K [67]) and residues with positively charged side chains predicted to interact with LMWH (K16, K28) [68] were also included. Neutral residues in regions distant to the proposed heparin binding site were substituted as controls (V18A, M35A, V36A; Table S3). In total, residues that are located on the 3Q fibril apices, on the fibril sides, and distant from the proposed binding site were assessed, providing good coverage across the fibril structure. In each case, the ability of the variant Aβ40 monomer to elongate 3Q fibril seeds was verified by ThT fluorescence (Figs. 4a and S4). Although differences in absolute fluorescence intensity were detected between Aβ40 variants presumably due to small structural changes that affect ThT binding, all variants except M35A (Fig. S4N) showed the ability to be seeded with 3Q fibrils and were further analyzed for GAG binding. TEM verified the presence of long, straight fibrils in all samples following seeding (Figs. 4b and S5). To further confirm the presence of 3Q morphology, SSNMR was performed on a subset of variants (H6F, E22K, I31T), all of which showed a characteristic cross peak attributed to the close proximity of H13 and V40 side chains in the hairpin structure and also detected in WT 3Q fibrils ([45], Fig. S6). The binding affinity of LMWH for each fibril sequence was next analyzed as described above. The results (Figs. 4c and 5a–c, and S7; Table S4) showed that the 3Q variant fibrils bind LMWH, with affinities that span values similar to WT 3Q (S26A, Fig. 4c-iv; H14F, Fig. S7E; V36A, Fig. S7 K; ΔΔ*G*° binding < 1.1 kJ/mol), to variants with affinities that are reduced (R5A, Fig. S7C; H6F, Fig. 4c-i; A21G, Fig. S7F; ΔΔ*G*° binding > 2.2 kJ/mol; Fig. 5b, Table S4). The weak-binding residues comprise the disordered N-terminal region in the 3Q fibril structure and, with residues K28 and I31 that lie at the start of β-strand 2 (K28A, Fig. S7I; I31T, Fig. S7 J; ΔΔ*G*° binding ~ 2.0 kJ/mol), form the “corners” of the triangular fibril topology that have been predicted previously based on chemical shift perturbations to provide the epicenter of LMWH binding [37], [49] (Fig. 5a–c). Residues H13–K16 located in β-strand 1 form a positive “stripe” on the fibril exterior that has been proposed to bind heparin [68]. However, these residues show only moderate changes in ΔΔ*G*° binding (1.1–1.9 kJ/mol) when substituted individually with neutral residues (H13F, H14F, K16A; Fig. 4c-ii and iii, Fig. S7E), ruling out interaction of LMWH with the sides of the 3Q fibrils as the dominant binding surface. Interestingly, residue V36A, which is distant from the proposed binding site (Figs. S7K and 5b–c) also exhibits a small, but significant ΔΔ*G*° binding (1.1 kJ/mol). This may reflect minor changes in fibril structure during elongation from WT 3Q seeds. Most notably, substitutions in N-terminal residues (A2T, R5A, H6F, D7N) result in > 2 kJ/mol ΔΔ*G*° binding (Fig. 5b–c), supporting the view that GAG–3Q binding involves a specific interface at the fibril “corners,” while other positively charged regions, such as the “stripe” along the fibril axis formed by the parallel in register stacking of H14 and K16, play a relatively minor role. These results suggest that 3Q–LMWH binding does not simply result from non-specific electrostatic complementarity, but instead relies on precise alignment of positively charged residues with specific sulfate moieties in the GAG itself.

## Discussion

### Origins of a specific LMWH–3Q fibril interaction

The results described above provide a rationale for the ubiquitous presence of GAGs in amyloid deposits and reveal that the interaction between LMWH and 3Q fibrils displays a specificity that extends beyond a simple electrostatic model, involving precise interactions between specific sulfates on the GAG and positively charged residues on the fibril surface (the “corners” of 3Q fibrils). This cluster of positive charges in the amyloid fibril structure is absent in Aβ40 2A fibrils and other fibril classes with linear arrangements of β-strands, such as Aβ16–22, amylin, and the recently described structures of Aβ42 fibrils [69], [70], rationalizing the previously identified lower binding affinity of LMWH for these fibril types (Fig. S1) [37]. Such precise surface complementarity may explain the fundamentally different binding affinity of GAGs for amyloid fibrils formed from different protein sequences and even for different fibril structures formed from identical or very similar protein sequence variants (Fig. S1) [37], [49].

A heparin binding site has been mapped previously on Aβ40 fibrils formed *de novo,* which contains a mixtures of different fibril structures, but nonetheless, it was shown to include residues H13, H14, and K16 [68]. Despite the presumably optimal location of H14 or K16 on the 3Q fibril “sides,” which produces a “stripe” of positive charge along the fibril axis seeming ideal for binding negatively charged GAGs, independent individual substitutions of H13, H14, and K16 in 3Q fibrils did not eliminate LMWH binding, suggesting that the “corners” of 3Q fibrils provide a binding site more optimized for interaction with LMWH. In accord with these findings, a recent survey of heparin binding proteins [40] revealed that there is no “universal” heparin binding motif as such. Rather, short points on the protein surface comprising typically either a positively charged or hydrogen bonding amino acid adjacent to a hydrophobic residue, distributed throughout a particular protein sequence, act as GAG–protein interaction sites, emphasizing the importance of structure in protein–heparin binding. By characterizing variants of both the heparin molecule and protein sequence for the interaction between 3Q fibrils and LMWH, we show here that binding is dominated by specific residues and substituents on the GAG, notably residues at the 3Q symmetry axis of the fibril and the 6-OS position of the polysaccharide. The 6-OS group, shown here to be an important component of GAG binding to Aβ40 3Q fibrils, has also been demonstrated as a sulfate-dependent regulator of the BACE-1 enzyme, an upstream effector of Aβ production [71]. Hence, as 6-deOS is a poor inhibitor of Aβ production relative to 6-OS heparin, it exerts a double effect on the aggregation cascade: enhancing Aβ formation and limiting heparin binding to the resultant fibrils. Thus, the importance of the 6-OS sulfate group in the GAG has far-reaching consequences that may affect AD manifestation and progression.

While the fibril types studied here are *in vitro*-derived structures, fibrils with similar three-fold symmetric morphologies have been found *in vivo* [72] by elongation of plaque material taken from the brain of a patient with AD with monomeric Aβ40. This modeled structure is also 3-fold symmetric and contains a cluster of charged amino acids involving residues R5-D7 and S26-K28. From the experiments presented here, we predict that this brain-derived fibril morphology should also bind GAGs with an affinity similar to that of 3Q fibrils. Recently, Qiang and colleagues [41] characterized a more extensive array of amyloid fibrils elongated from plaques in patients with AD who manifested different disease progression rates, symptoms, and plaque localizations. The resulting SSNMR spectra of these samples extended using Aβ40 or Aβ42 monomers showed several dominant fibril morphologies, suggesting that fibril formation *in vivo* may result in a limited number of fibril structures that differ in individuals with different disease types and manifestations, paralleling other reports of the analysis of amyloid extracted from human tissue [48]. Interestingly, several of the dominant Aβ fibril structures observed *in vivo* have chemical shifts comparable to those of Aβ40 3Q amyloid fibrils, providing a link between the binding of GAGs to 3Q fibrils produced *in vitro* described here to amyloid plaques within patients. The results presented also indicate that not all amyloid fibrils will bind heparin or partially desulfated heparin derivatives with equal affinity, potentially explaining the different GAG content in fibril plaques and different disease progression between individuals [47], [72]. It will be interesting to see in the future whether different GAG molecules co-localize within different amyloid plaques and whether GAG content correlates with disease phenotype and/or disease progression.

**Fig. 6:**
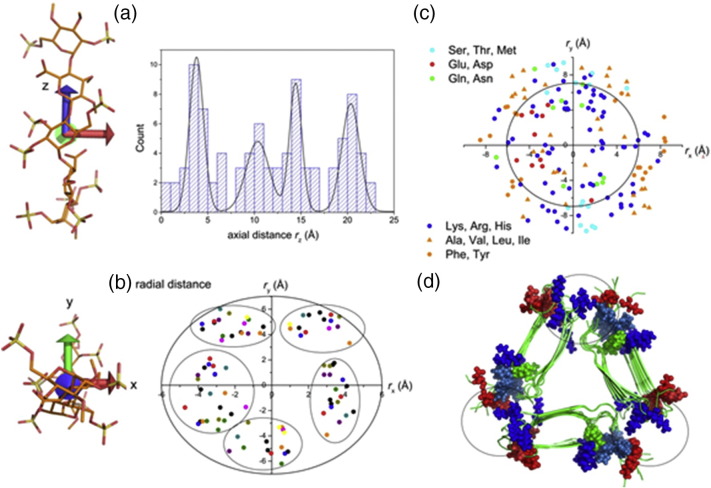
Rules for heparin–fibril binding. Crystal structures of 16 unrelated proteins (Table S5) containing bound heparin fragments ranging from dimers to hexamers were compiled and any extraneous molecules were removed, leaving the protein and heparin. The structures were then rotated into a new reference frame corresponding to the principal axes of inertia of heparin in each case, with the origin at the center of mass of heparin. (a) The distribution of sulfate groups along the *z*-axis. (b) Cross-section view of the heparin molecules in which each point represents the position of a sulfur atom. (c) The positions of amino acid residues within 4 Å of one or more sulfate groups. (d) The structure of 3Q fibrils (2LMQ [45]) with acidic and basic residues shown in red and blue, respectively. The circles represent the cross-sectional space of heparin.

Additional clues about the nature of the compatibility between heparin and Aβ40 were gleaned from the analysis of 16 unrelated crystal structures of protein–heparin complexes, in which the GAG conformation and the proximity of the sulfate groups to different residue types were examined (Fig. 6, Table S5). All GAG ligands examined adopted an approximately linear structure at the protein surface with sulfate groups distributed along the principal (*z*) axis of inertia with a separation of around 5 Å (Fig. 6a). This separation mirrors the ~ 4.7-Å repeating distance along the fibril long axis, and so it is reasonable to propose that in the LMWH–3Q complex, the GAG orientation is approximately parallel to the fibril axis. When viewed down the principal axis, the GAG ligands occupy a cross-sectional area of 130 Å^2^, and the sulfate groups cluster into five radial positions (Fig. 6b). Hence heparin is capable of forming ionic interactions with multiple protein surfaces, and indeed, such a multi-faceted interaction is expected to confer extra stability on the complex. Acidic and basic residues within Aβ are in closest contact with the ligands and extend into the cross-sectional space and typically within 4 Å of one or more sulfate groups, whereas non-polar/aromatic residues and Thr, Ser, and Met tend to be more peripheral (Fig. 6c). Figure 6d illustrates the cross-sectional area of heparin when mapped onto the heparin binding site at the apices of the 3-fold symmetric 3Q fibril structure. The model implies that heparin can interact simultaneously with an interface formed by the N- and C-termini of one monomer and a second interface formed by the loop region of an adjacent monomer. Furthermore, the interaction site within the heparin cross-sectional space is occupied by acidic and basic residues, allowing intimate contact with the sulfate groups with polar hydrogen bonding residues situated peripherally. Together, these features may explain the uniquely high affinity of heparin for the 3Q morphology while, at the same time, rationalizing the ubiquity of GAG–amyloid interactions observed *in vitro* and *in vivo* [56], [73].

A recent review by Kisilevsky *et al*. [74] posits that one outstanding question in the amyloid field is whether GAGs and other co-precipitating plaque components are “associated factors” or “critical components” of aggregation. Previous studies, along with the work presented here, indicate that while GAGs are not required for fibrillation of Aβ40 *in vitro*, they alter the kinetics of amyloid fibril growth [10], [17], [21], [23], stabilize fibrils [24], [25], alter toxicity [52], [73], and bind extensively and specifically to the fibril surface [37], [49], potentially competing for interaction with molecular chaperones [75], [76], metal ions [6], [77], nucleic acids [78], and other components known to associate with amyloid fibrils and to affect amyloid formation [79], [80]. GAGs should thus perhaps be considered as “influential participants” in Aβ40 aggregation and deposition. In summary, the results presented suggest a topologically focused model for GAG–amyloid interactions and indicate that the structural polymorphism exhibited by amyloid fibrils both *in vitro* [45], [55] and *in vivo* [41], [72] may affect the interactions of fibrils with key accessory molecules. These co-factors, as a result, may play a significant role in determining disease development, presentation, and progression.

## Materials and Methods

### Expression and purification of Aβ40 and its variants

Commercial *E. coli* strain BL21 (DE3) cells (Agilent) were transformed with a pETSAC plasmid containing the sequence for Aβ40 [54], and bacteria were grown in minimal medium for ^13^C, ^15^N-labeled samples [37] or LB media for unlabeled samples [54]. Cultures were grown and Aβ40 purified as described previously [37], [49]. An identical procedure was used for purification of both labeled and unlabeled samples. The resulting Aβ40 protein contains an additional N-terminal methionine residue that has no effect on the fibrillation of Aβ40 or the morphology of fibrils formed [54]. Final protein concentrations were estimated from UV absorption in 7 M guanidinium chloride at 280 nm using an extinction coefficient of 1490 M^− 1^ cm^− 1^. Site-directed mutagenesis was performed using a Q5 kit (NEB) to create each variant of Aβ40. Substitutions were verified by plasmid sequencing (Beckman-Coulter Genomics). All variants were expressed and purified as described for the WT sequence.

### Aβ40 3Q fibril preparation

3Q fibril seeds of Aβ40 were prepared by diluting fibrils of the 3Q morphology [45] (a kind gift from R. Tycko) to 5% (v/v) in seeding buffer [25 mM NaH_2_PO_4_ (pH 7.5), 0.01% (w/v) NaN_3_] and sonicating for 5 s “on,” 45 s “off” for 3 cycles at amplitude 20% (approx. 3 J) to produce seeds. Lyophilized monomeric Aβ40 (or variant) was added to the fibril seeds to a concentration of 0.9 mg/mL and incubated quiescently overnight at 25 °C. After 18 h, the fibrils were sonicated for 5 s and incubated quiescently at 25 °C for 1 week*.* Fibril growth was verified using negative stain TEM.

### Seeded fibril growth kinetics monitored by ThT fluorescence

To ensure that 3Q fibrils were forming by seeded elongation of 3Q fibril seeds, fibril growth was monitored using 20 μM Aβ40 monomer in the presence or absence of 5% (v/v) 3Q seeds in seeding buffer containing 10 μM ThT. Samples were incubated quiescently at 37 °C in a 96-well plate (Corning 3881) sealed with Star Seal polyolefin film (StarLabs) on a Fluorostar OPTIMA plate reader (BMG Labtech). Fluorescence was monitored continuously at an excitation wavelength of 440 nm and emission wavelength of 480 nm for a minimum of 3 days. Only samples that clearly demonstrated seeded growth and showed no evidence for spontaneous (unseeded) assembly were taken forward for analysis of heparin binding. E22 variants, which formed fibrils rapidly under unseeded conditions, showed difference fluorescence intensity and curve shape in unseeded *versus* seeded samples as shown.

### TEM

A 10-μL drop of fibril sample was applied to a formvar/carbon-coated copper specimen grid (Agar Scientific Ltd., Stansted, UK) The drop was blotted with filter paper after 30-s incubation. The grid was then washed with 2 × 10 μL of water and 10 μL of 2% (w/v) aqueous uranyl acetate. A second drop of 10 μL of 2% uranyl acetate was then applied to the grid and incubated 30 s and then dried at room temperature. Grids were examined in a JEOL JEM-1400 transmission electron microscope in the Astbury Biostructure Laboratory.

### SSNMR experiments

Following 1 week of quiescent growth at 25 °C, 5 mg total of 0.9 mg/mL fibrils was pelleted by centrifugation at 50,000*g* for 1 h in an ultracentrifuge (Beckman Coulter) and the supernatant was removed. The pellet was packed in its hydrated state into a 3.2-mm zirconia MAS rotor without further treatment for analysis by SSNMR. 2D ^13^C–^13^C spectra were recorded at 16.3 T with a 3.2-mm HXY probe operating in double-resonance mode and magic-angle spinning at 14 kHz. The operating temperature was 4 °C. Hartmann–Hahn cross-polarization was achieved with a 2-ms contact time, and 100-kHz proton decoupling with SPINAL-64 was applied during signal acquisition. Spectra were recorded with either a 10-ms or 50-ms mixing time during which the proton nutation frequency was adjusted to the MAS frequency of 14 kHz to meet the DARR condition [81]. Typically, 480 increments were acquired in the indirect (*t*_1_) dimension with 400–600 transients per increment, and the total measurement time varied from 2 to 7 days depending on the efficiency of rotor packing. Phase-sensitive detection in the indirect dimensions was achieved using the States-TPPI method. Chemical shifts are expressed relative to tetramethylsilane. The simulated ^13^C–^13^C spectrum based on previously published chemical shifts was obtained using a C program written specifically for this purpose.

### Preparation of heparin molecules with specific substitutions

Modified heparin derivatives were prepared and characterized as described [57]. *N*-trifluoroacetyl heparin was prepared from deNS heparin as reported and sized *via* gel filtration [82]. Unmodified LMWH (approx. dp18) was also purchased from Iduron (Cheshire, UK). Purchased LMWH had similar binding properties to 3Q fibrils as in-house preparations of heparin of comparable length [compare Fig. 2c-i (produced in-house) and Fig. S7A (purchased)].

### Quantitation of heparin binding to 3Q fibrils

*In vitro* binding assays of fibril–heparin binding were performed by quantifying the amount of GAG remaining unbound at different fibril–GAG concentration ratios, as described previously [49]. In brief, fibrils (25 μL of a monomer equivalent concentration of 0.9 mg/mL) were pelleted by centrifugation at 14,000*g* for 30 min to separate fibrillar material from residual monomers. The pellet containing the fibrils was then resuspended in a 1- to 10-fold molar excess of heparin in seeding buffer in a total final volume of 125 μL. An LMWH mass of 4650 Da was assumed for calculations of saccharide concentration. The sample was incubated at room temperature quiescently overnight. Samples were then centrifuged at 14,000*g* for 30 min to pellet the fibrils and associated GAG, and the supernatant was removed and placed in a clean Eppendorf tube and assayed for heparin content. Saccharide concentration was quantified by the addition of 25 μL of heparinase II (produced in-house, according to Ref. [83]) in 20 mM Tris–HCl, 50 mM NaCl, 4 mM CaCl_2_, and 0.01% (w/v) BSA (pH 7.5), which cleaves the glycosidic bond generating unsaturated uronic acid. The reaction was incubated on a rotator at room temperature for 18 h and quenched by the addition of 850 μL of 50 mM HCl. Samples were prepared in parallel with heparin alone (no fibril) as standards so as to account for any differences in *k*_cat_ of heparinase II for different GAG substrates [62]. The uronic acid content was then determined by measuring the absorbance at 232 nm using an extinction coefficient of 5500 M^− 1^ cm^− 1^ [84]. Three replicates were assayed in parallel, and bound heparin was determined by comparison to replicates of heparin cleavage in the absence of fibrils. Fibril alone (no GAG) and pellet washes were also included in each set. An identical procedure was used to quantify GAG binding to fibril variants and for the heparin variants analyzed here. Binding curves were fitted to the Hill equation:

(1) $$\theta ={\left[L\right]}^{n}/\left(K_{d}+{\left[L\right]}^{n}\right)$$

where *θ* is the fraction of Aβ bound to heparin, [*L*] is the concentration of unbound heparin, *n* is Hill coefficient (cooperative sites), and *K*_d_ is the dissociation constant. Each protein variant and modified heparin variant was first fitted individually. For all variants, the Hill coefficient fitted in this way was 3.0 ± 0.3. This value was then held constant, and the data were refit over all variants to yield the *K*_d_ values presented in all figures and tables. From the difference in *K*_d_ values of GAG binding to WT and variant fibrils, or the binding of LMWH and its derivatives, ΔΔ*G*° binding was calculated from:

(2) $$\begin{array}{l} {\mathrm{\Delta G}}^{{}^{\circ}}=\mathrm{RT}\ln\left(K_{d}\right)\\ {\mathrm{\Delta \Delta G}}^{{}^{\circ}}=\left({\mathrm{\Delta G}}^{{}^{\circ}}-{\mathrm{\Delta G}}^{{}^{\circ}}\mathrm{WT}\right) \end{array}$$

where *R* is the universal gas constant (8.315 J/mol K), *T* is temperature (298 K), and *K*_d_ is calculated from Eq. (1) to yield an apparent free energy of binding Δ*G*°. This value is then subtracted from the free energy of binding from WT 3Q fibrils to LMWH (or dp18) for the change in apparent free energy of binding, ΔΔ*G*°.

### Molecular modeling

Using the structural model of residues 9–40 previously reported in 3Q fibrils (2lmq.pdb [45]) as a starting point, a model of full-length Aβ40 was constructed using Modeller v. 9.13 [85]. Scripts were written to add the flexible N-terminal residues 1–8 missing from 2LMQ, preceded by an additional Met produced by recombinant expression of the peptide. Analysis of heparin–fibril complexes was performed using a C program written specifically for the purpose.

## Appendix A Supplementary data

Supplementary material Image 2
