# Supplementary material for: Molecular Origins of the Compatibility between Glycosaminoglycans and Aβ40 Amyloid Fibrils
Source: J Mol Biol. 2017 Aug 4;429(16):2449–62. doi: 10.1016/j.jmb.2017.07.003 (PMC5548265; doi:10.1016/j.jmb.2017.07.003)
Supplement: Supplementary file 1 — Supplementary material [file mmc1.docx]

**Supplemental Material**

**Molecular Origins of the Compatibility Between Glycosaminoglycans and Aβ40 Amyloid Fibrils**

Katie L. Stewart^±^, Eleri Hughes^‡^, Edwin A. Yates^≈^, David A. Middleton^‡*^and Sheena E. Radford^±*^

*^±^Astbury Centre for Structural Molecular Biology, School of Molecular and Cellular Biology, University of Leeds, Leeds LS2 9JT, UK*

*^‡^Department of Chemistry, University of Lancaster, Lancaster LA1 4YB, UK*

*^≈^Department of Biochemistry, Institute of Integrative Biology, University of Liverpool, Liverpool L69 7ZB, UK*

Contents:

Figure S1: LMWH binds most tightly to Aβ40 fibrils with the 3Q morphology.

Figure S2: 2D ^13^C-^13^C DARR spectra of Aβ40 3Q fibrils.

Figure S3: Binding of 3Q fibrils to modified heparin constructs.

Figure S4: Seeded elongation assays showing that Aβ40 variants can be seeded with 3Q seeds formed from the wild-type (WT) protein.

Figure S5: TEM images of fibrils formed by addition of different Aβ40 variants to wild-type 3Q fibril seeds.

Figure S6: SSNMR spectra of Aβ40 variants showing crosspeaks characteristic of 3Q fibrils.

Figure S7: Binding of 3Q fibril variants to LMWH.

Table S1: Chemical shift assignments for wild-type 3Q fibrils, from Figure S2.

Table S2: Summary of binding energies of modified heparin moelcules to 3Q fibrils.

Table S3: Summary of Aβ40 variants characterized.

Table S4: Summary of binding energies of LMWH to Aβ40 3Q variants.

Table S5: Summary of the X-ray crystal structures of protein-heparin fragment complexes used in the computation analysis in Figure 6 of the main text.

**Supplemental Figures**


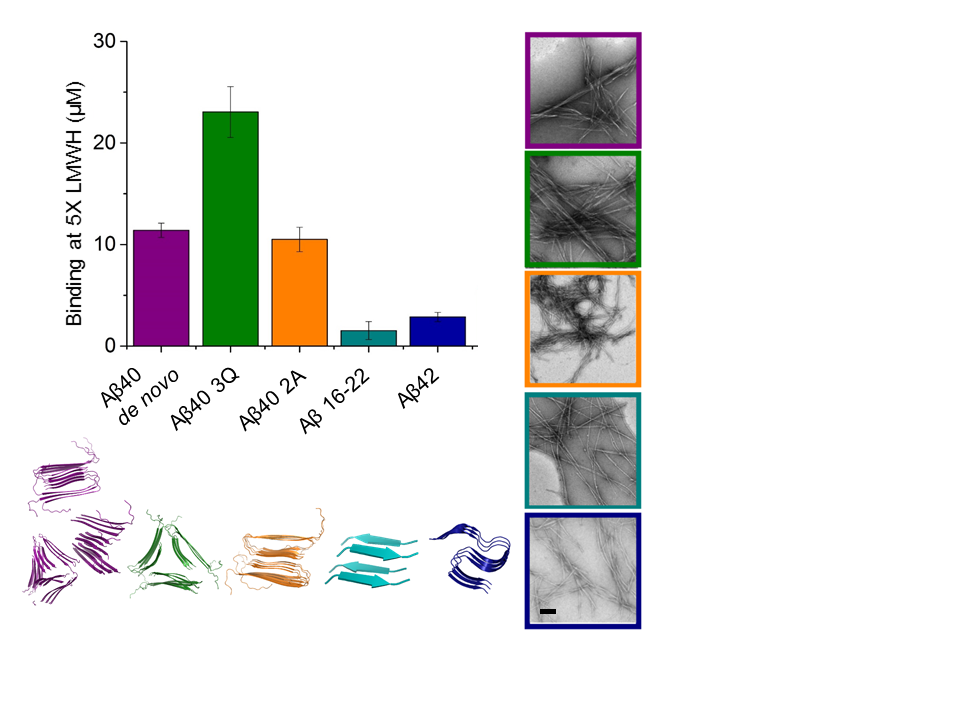
**Figure S1**

**Figure S1**: LMWH binds most tightly to Aβ40 fibrils with the 3Q morphology. This figure is re-drawn from reference [1]. Here, the amount of LMWH bound to 25 µM fibrils of each type (monomer equivalent concentration) when LMWH is added to five-fold molar excess is plotted, with standard deviation from 3 replicate assays. *De novo* fibrils are structurally heterogeneous and produced without seeding. 3Q and 2A fibrils were formed by elongation of each seed type (kindly provided by Rob Tycko [2]) with Aβ40 monomers (Methods). Aβ42 fibrils were prepared to replicate a homogenous morphology recently described [3]. Structures shown are based on PDB ID codes 3Q: 2LMQ [2], 2A: 2LMN [4], 16-22: built from 3OW9 [5], Aβ42: 2MXU [6], colored as in the bar chart. Negative stain EM images of the fibrils formed are shown, colored as in other panels. The scale bar is 200 nm and is the same for all images.

**Figure S2**

**
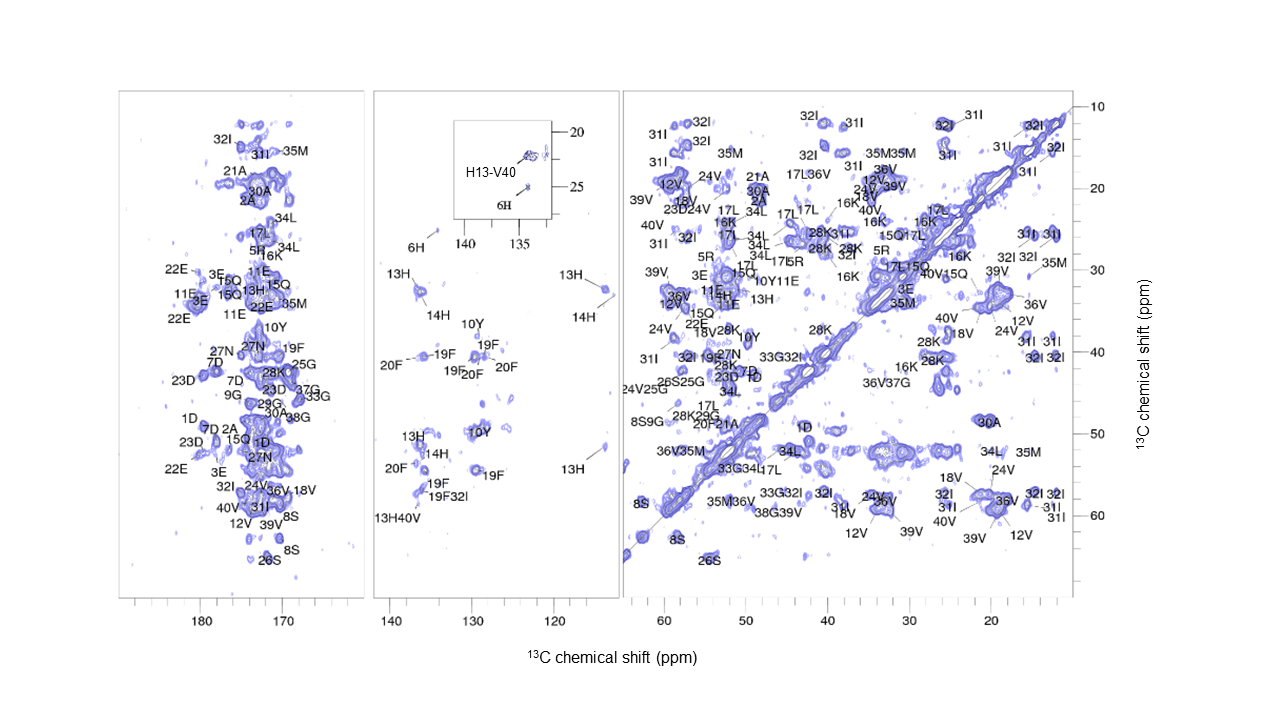
**

**Figure S2**: 2D ^13^C-^13^C DARR spectra of Aβ40 3Q fibrils. 2D ^13^C-^13^C spectrum of 3Q seeded fibrils obtained with 50 ms DARR mixing in the absence of LMWH. The inset shows the H13-V40 cross peak, indicative of the ‘hoop’ structure of 3Q fibrils [2], which was observed when the temperature was reduced to -20ºC.

**Figure S3**

**
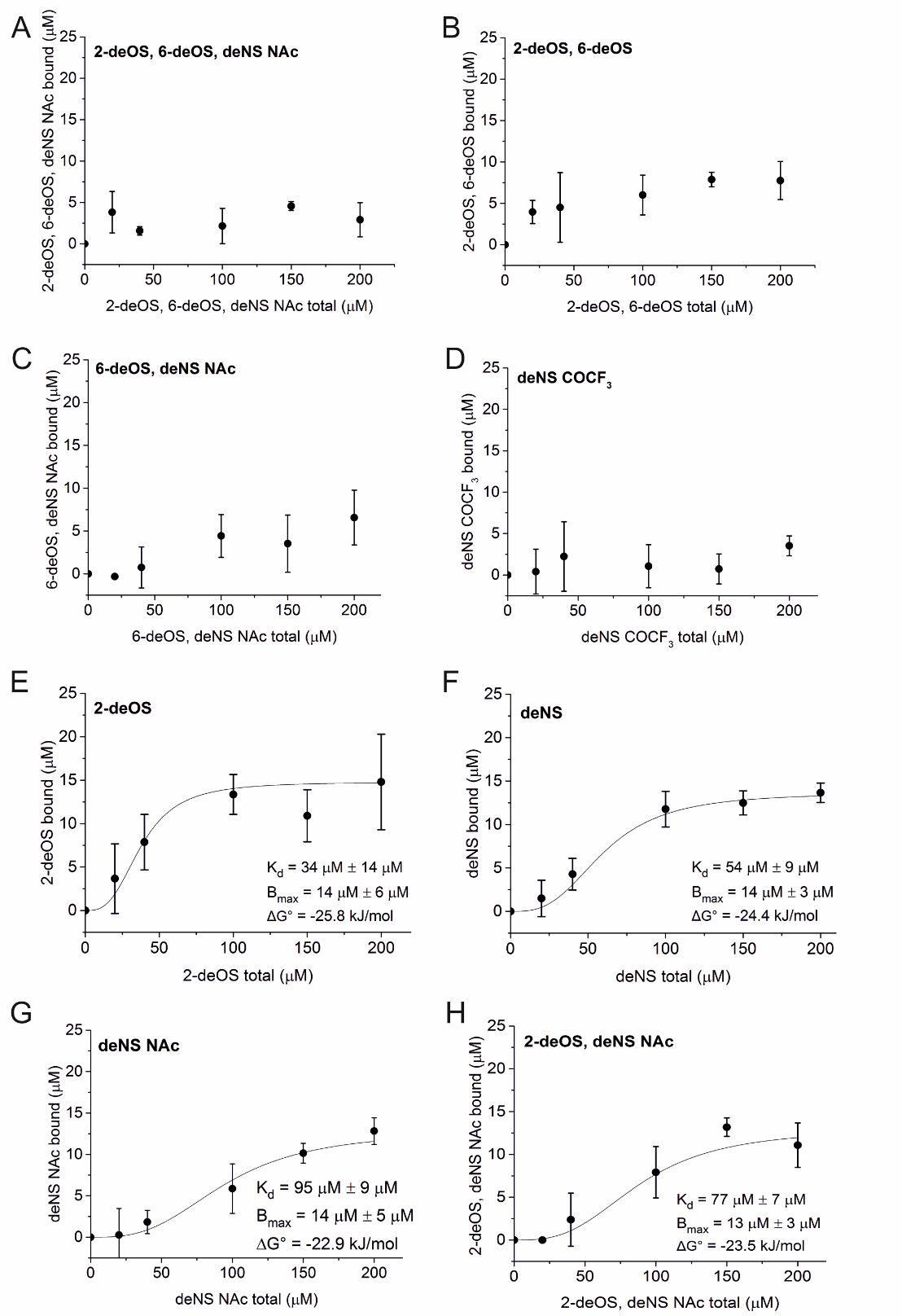
**

**Figure S3**: Binding of 3Q fibrils to modified heparin constructs. Each data point represents the average of three replicates at each heparin concentration, with standard deviation, corrected against GAG alone cleavage. B_max_, K_d_, and ΔG° binding values are reported where binding was detected. The solid line was obtained by non-linear least-squares fitting of a Hill function for all plotted data. The data are summarized in Table S2.

**Figure S4**


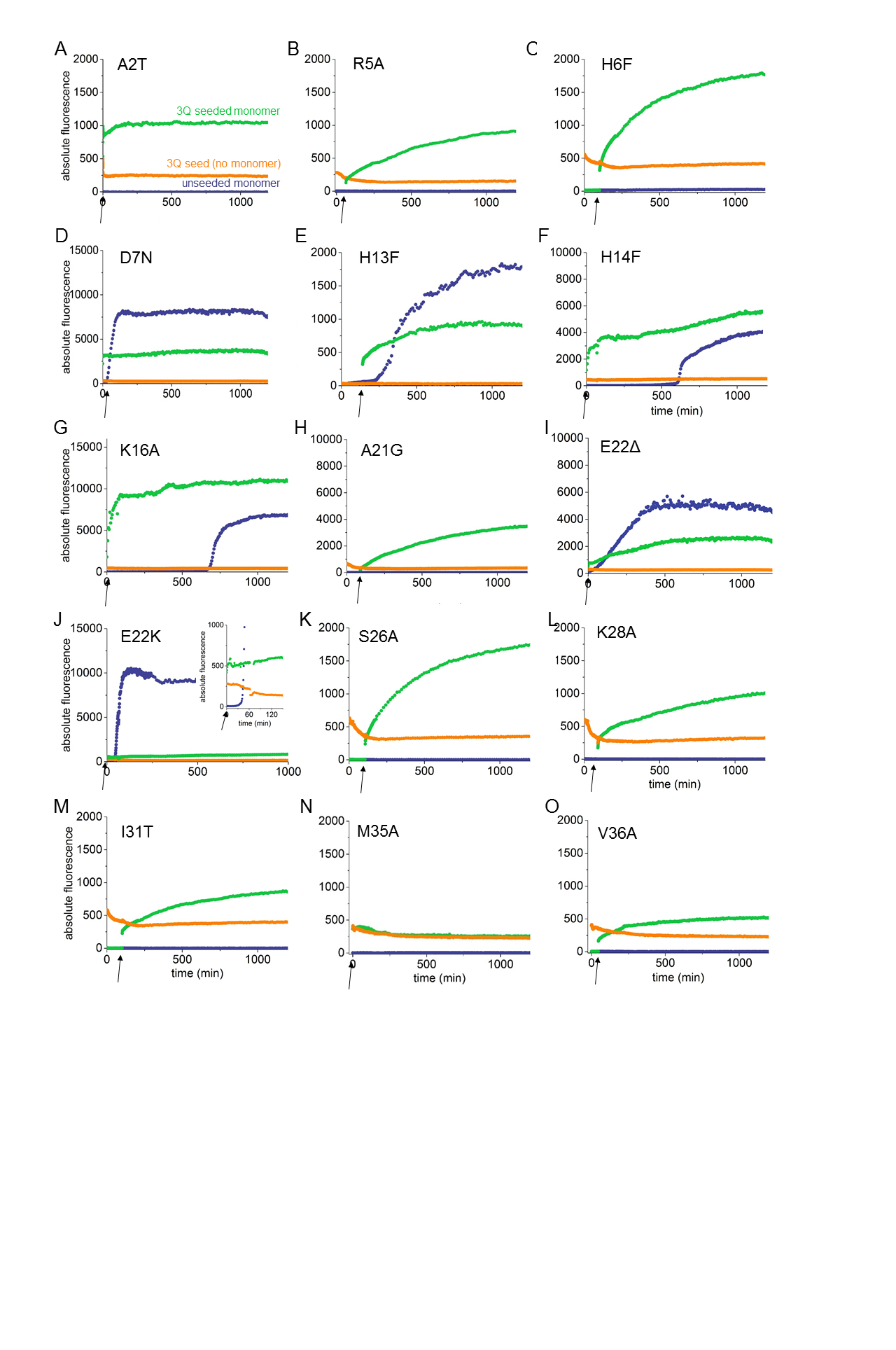


**Figure S4**: Seeded elongation assays show that all variants, except M35A, can be seeded with 3Q seeds formed from wild-type (WT) Aβ40. For each experiment 20 µM variant monomer was incubated with 5% (v/v) WT 3Q seeds quiescently in 25 mM sodium phosphate buffer, pH 7.5, 0.01% (w/v) azide at 37 ⁰C with 10 µM ThT. Seed was added to monomer at or near the start of incubation, as indicated by an arrow on each panel, causing a rapid increase in fluorescence (green). The 5% (v/v) seed alone (orange) and unseeded variant monomer (purple) samples were also measured. Each of the curves shown is a representative based on four replicates. Note that some unseeded variants (such as E22Δ and E22K) show rapid growth, but that the rate of aggregation and absolute fluorescence intensity differs from seeded samples, allowing spontaneous versus seeded growth profiles to be distinguished.

**Figure S5**


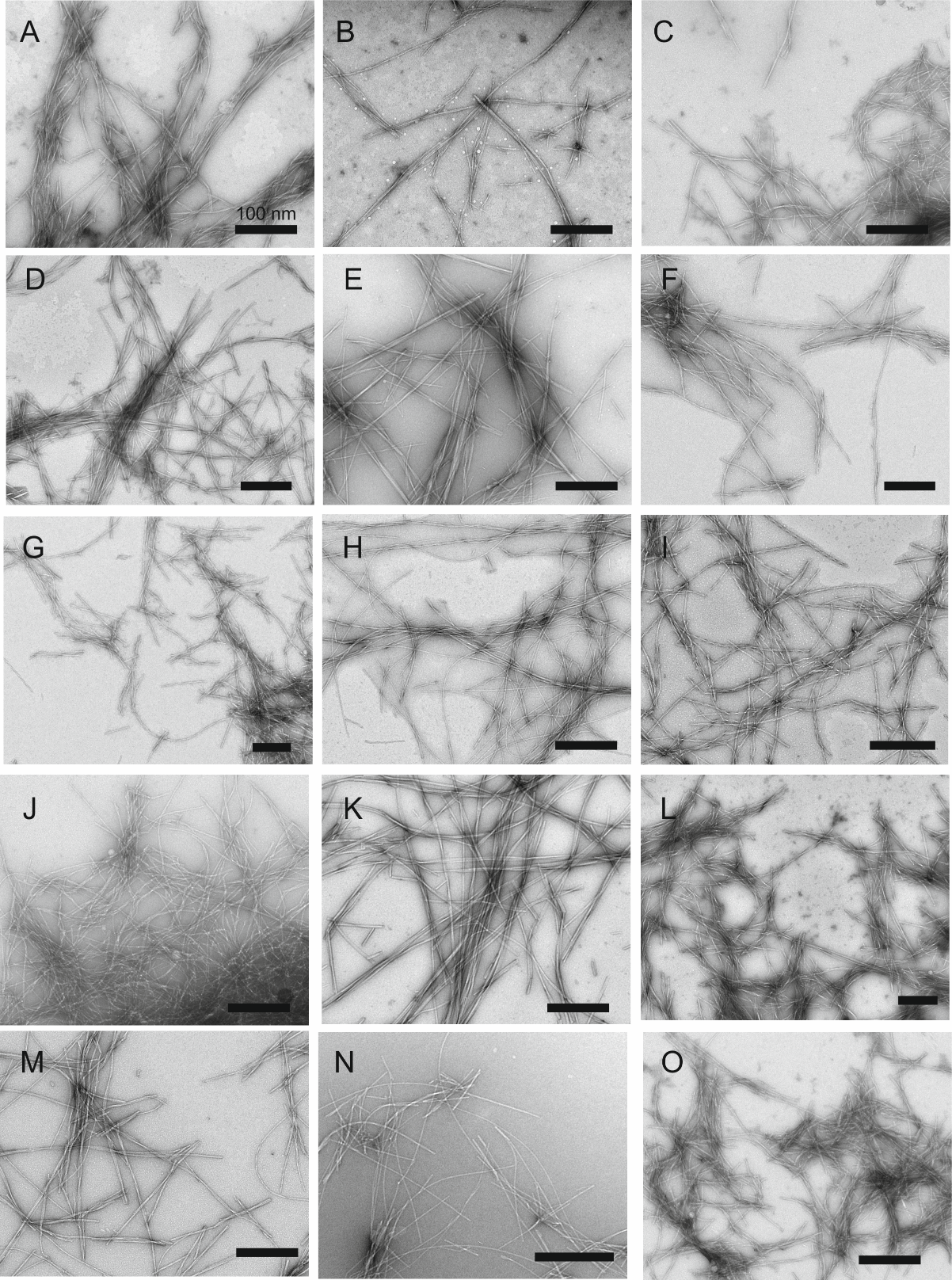


**Figure S5**: TEM images of fibrils formed by addition of different Aβ40 variants to wild-type 3Q fibril seeds. Fibrils formed from (A) WT; (B) A2T; (C) R5A; (D) H6F; (E) D7N; (F) H13F; (G) H14F; (H) K16A; (I) A21G; (J) E22Δ; (K) E22K (L) S26A (M) K28A (N) I31T (O) V36A Aβ40 monomers, each seeded with WT 3Q fibrils (5% (v/v), Figure S4) are shown. The scale bar is 100 nm in each panel.

**Figure S6**

**
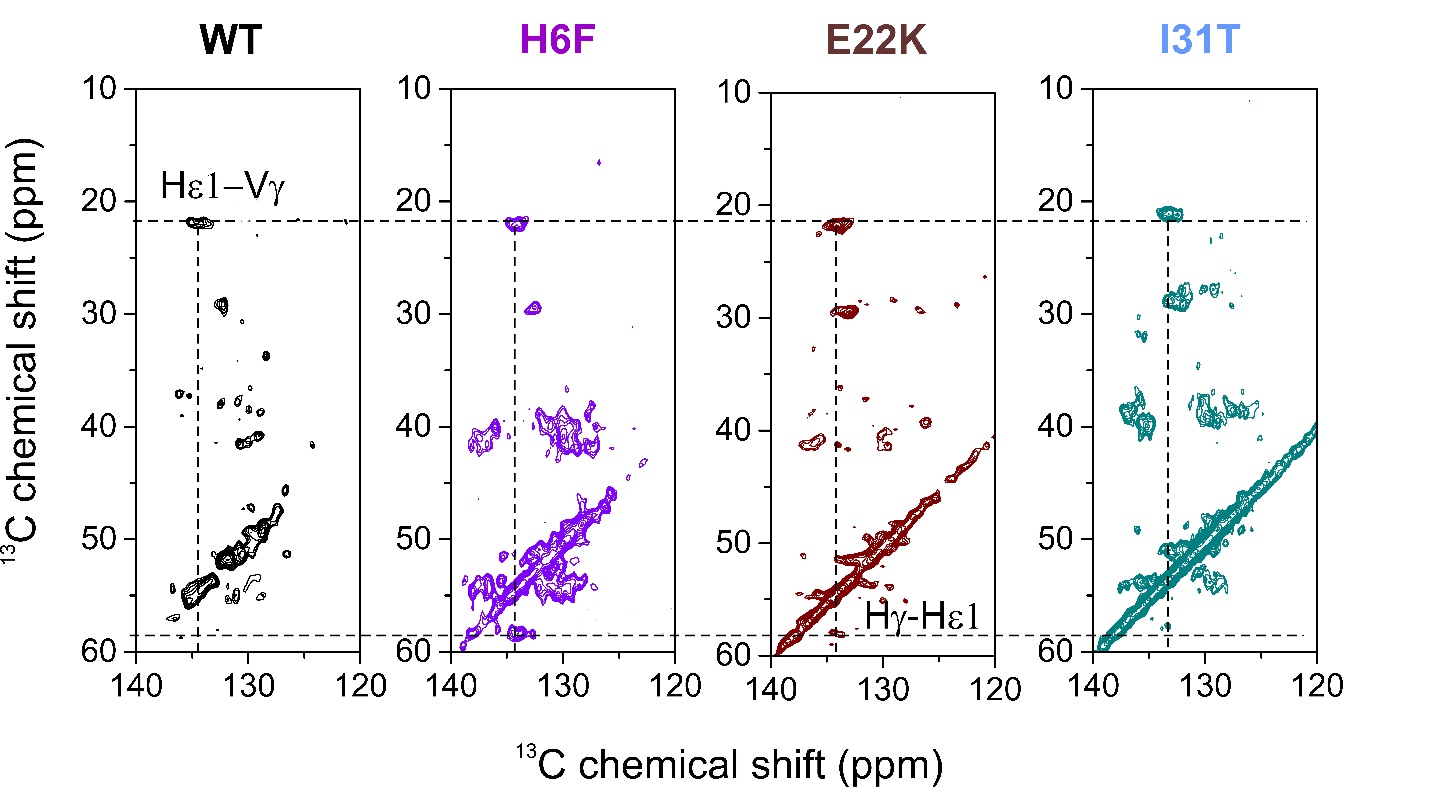
**

Figure S6: 2D ^13^C-^13^C SSNMR spectrum (with 50 ms DARR mixing) of WT and mutant fibrils showing characteristic cross-peaks attributed to cross-strand coupling between His and Val side chains, consistent with a hairpin structure.  Spectra were obtained at 16.3 T with magic-angle spinning at 14 kHz. All other conditions are as described in the main text.

**Figure S7**


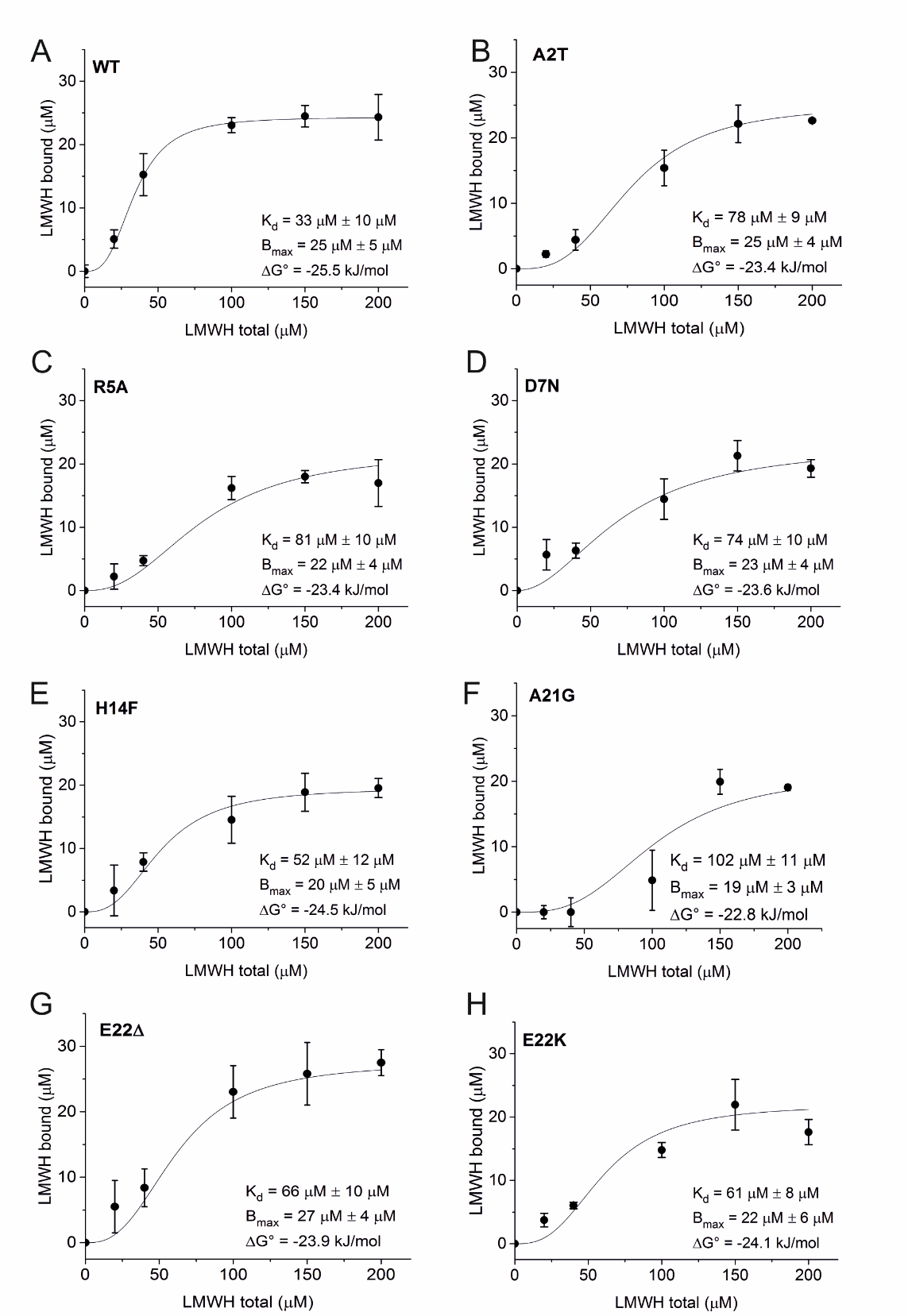


**Figure S7**: Binding of 3Q fibril variants to LMWH. Each data point represents three replicates with standard deviation at each LMWH concentration, assayed against LMWH alone samples. The solid line was obtained by non-linear least-squares fitting to a Hill function. The data are summarized in Table S4.

**Figure S7 (continued)**


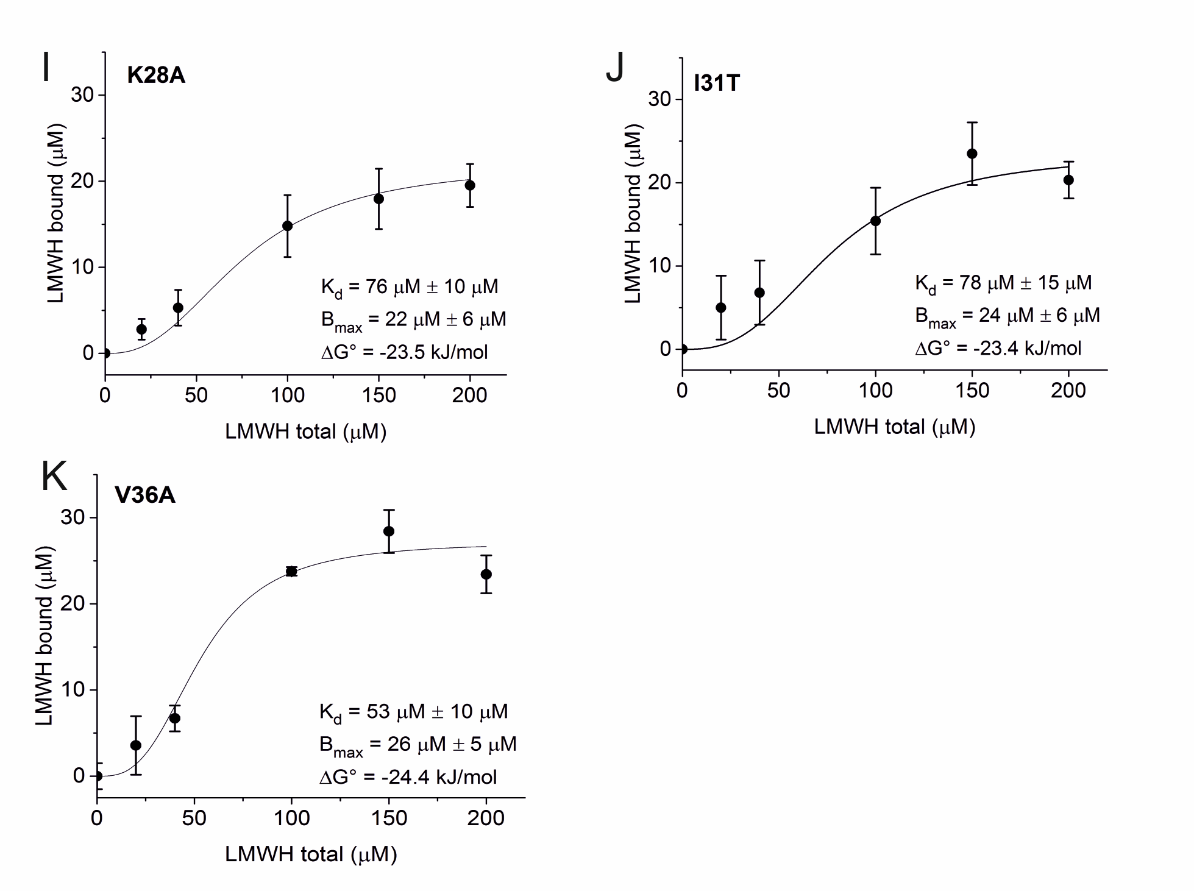


**Figure S7 (continued)**: Binding of 3Q fibril variants to LMWH Each data point represents three replicates with standard deviation at each LMWH concentration, assayed against LMWH alone samples. The solid line was obtained by non-linear least-squares fitting to a Hill function. The data are summarized in Table S4.

**Supplemental Tables**

**Table S1**

| **Residue** | **C** | **Cα** | **Cβ** | **Cγ** | **Cδ** | **Cε1** | **Cζ** |
| --- | --- | --- | --- | --- | --- | --- | --- |
| **D1** | 172.8 | 49.0 | 42.9 | 179.6 | -- | -- | -- |
| **A2** | 174.2 | 48.3 | 21.3 | -- | -- | -- | -- |
| **E3** | 178.0 | 52.6 | 30.3 | 32.0 | 180.0 | -- | -- |
| **F4** | -- | -- | -- | -- | -- | -- | -- |
| **R5** | 173.1 | 53.4 | 33.3 | 27.3 | 41.9 |  | 157.7 |
| **H6** | 171.6 | -- | 25.1 | -- | 117.4 | 134.2 | -- |
| **D7** | 173.8 | 50.8 | 42.3 | 178.0 | -- | -- | -- |
| **S8** | 170.3 | 58.3 | 62.7 | -- | -- | -- | -- |
| **G9** | 174.0 | 46.3 | -- | -- | -- | -- | -- |
| **Y10** | 172.7 | 49.6 | 38.1 | 129.1 | 131.4 | 114.8 | 155.0 |
| **E11** | 173.6 | 52.1 | 31.2 | 33.9 | 179.6 | -- | -- |
| **V12** | 173.6 | 59.2 | 33.9 | 19.2 | -- | -- | -- |
| **H13** | 173.7 | 51.4 | 32.3 | 130.3 | 113.7 | 136.6 | -- |
| **H14** | 171.2 | 53.3 | 32.9 | 129.4 | 112.4 | 136.3 | -- |
| **Q15** | 172.1 | 51.9 | 31.0 | 32.7 | 176.6 | -- | -- |
| **K16** | 171.5 | 52.5 | 34.2 | 23.8 | 28.0 | 40.1 | -- |
| **L17** | 172.6 | 52.1 | 42.4 | 26.5 | 22.4/25.4 | -- | -- |
| **V18** | 169.2 | 57.3 | 34.6 | 21.2 | -- | -- | -- |
| **F19** | 170.4 | 54.3 | 40.5 | 135.9 | 129.5 | 128.5 | 125.4 |
| **F20** | 171.4 | 53.6 | 40.6 | 136.7 | 128.6 | 127.9 | 125.4 |
| **A21** | 173.3 | 48.5 | 18.3 | -- | -- | -- | -- |
| **E22** | 172.6 | 52.6 | 30.2 | 34.3 | 180.3 | -- | -- |
| **D23** | 172.5 | 52.3 | 42.8 | 179.6 | -- | -- | -- |
| **V24** | 172.5 | 57.4 | 34.3 | 20.2 | -- | -- | -- |
| **G25** | 169.3 | 42.4 | -- | -- | -- | -- | -- |
| **S26** | 171.6 | 54.4 | 65.3 | -- | -- | -- | -- |
| **N27** | 172.9 | 52.0 | 40.4 | 175.0 | -- | -- | -- |
| **K28** | 172.9 | 52.1 | 37.1 | 25.1 | 27.1 | 40.8 | -- |
| **G29** | 171.6 | 46.1 | -- | -- | -- | -- | -- |
| **A30** | 172.8 | 48.4 | 20.1 | -- | -- | -- | -- |
| **I31** | 172.8 | 58.7 | 38.4 | 25.3/15.6 | 12.5 | -- | -- |
| **I32** | 175.0 | 57.1 | 40.4 | 25.7/14.7 | 12.0 | -- | -- |
| **G33** | 167.9 | 45.7 | -- | -- | -- | -- | -- |
| **L34** | 171.4 | 51.8 | 44.6 | 26.4 | 19.9/24.3 | -- | -- |
| **M35** | 170.7 | 51.9 | 33.6 | 30.7 | -- | 15.4 | -- |
| **V36** | 171.2 | 58.0 | 32.9 | 18.0 | -- | -- | -- |
| **G37** | 170.0 | 46.0 | -- | -- | -- | -- | -- |
| **G38** | 169.0 | 43.6 | -- | -- | -- | -- | -- |
| **V39** | 172.5 | 59.3 | 32.6 | 19.0 | -- | -- | -- |
| **V40** | 174.1 | 58.4 | 34.6 | 21.6 | -- | -- | -- |

**Table S1**: Chemical shift assignments for wild-type 3Q fibrils, from Figure S2. Additions to the published 3Q fibril structure assignments [2] are shown in blue. Chemical shifts were measured relative to tetramethylsilane (TMS) [7].

**Table S2**


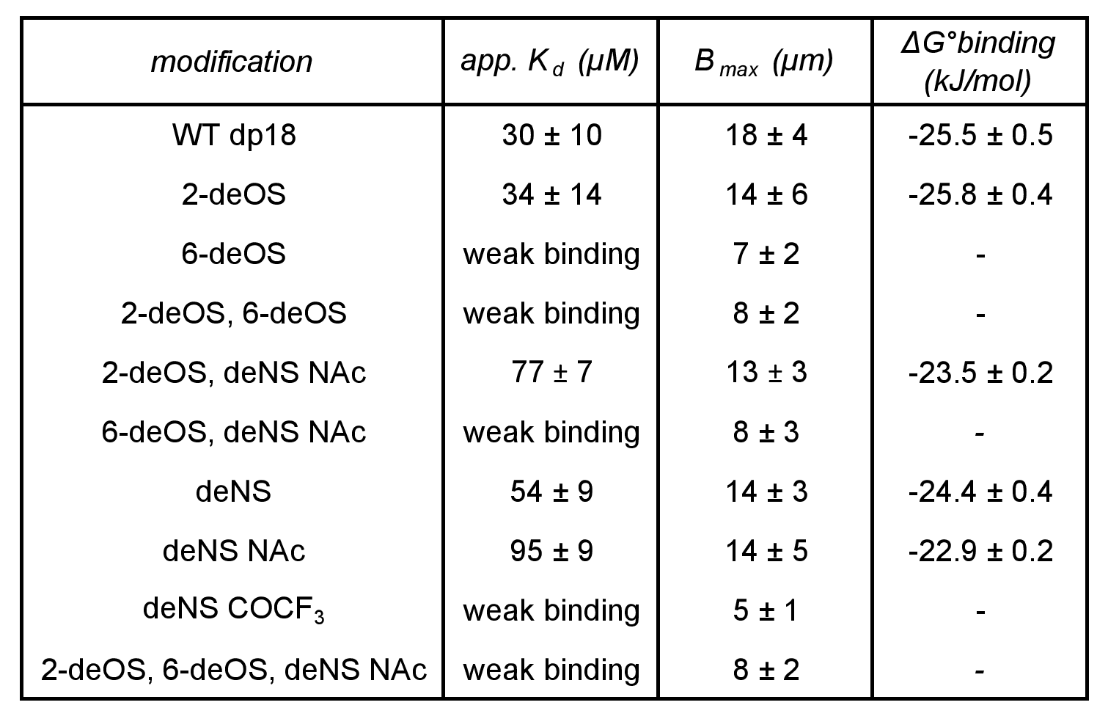


**Table S2**: Summary of binding energies of modified heparin moelcules to 3Q fibrils. Individual binding curves are shown in Figures 2 and S3 and determination of K_d_, B_max_, and ΔG° binding values (with standard deviation) for each molecule are described in the text and Methods. ‘Weak binding’ indicates that a K_d_ could not be determined over the concentration range of GAG employed (1- to 10-fold molar excess of GAG over Aβ concentration; a monomer equivalent concentration of 25 µM was used). In the latter cases, B_max_ describes the maximum binding at the highest concentration of GAG employed.

**Table S3**


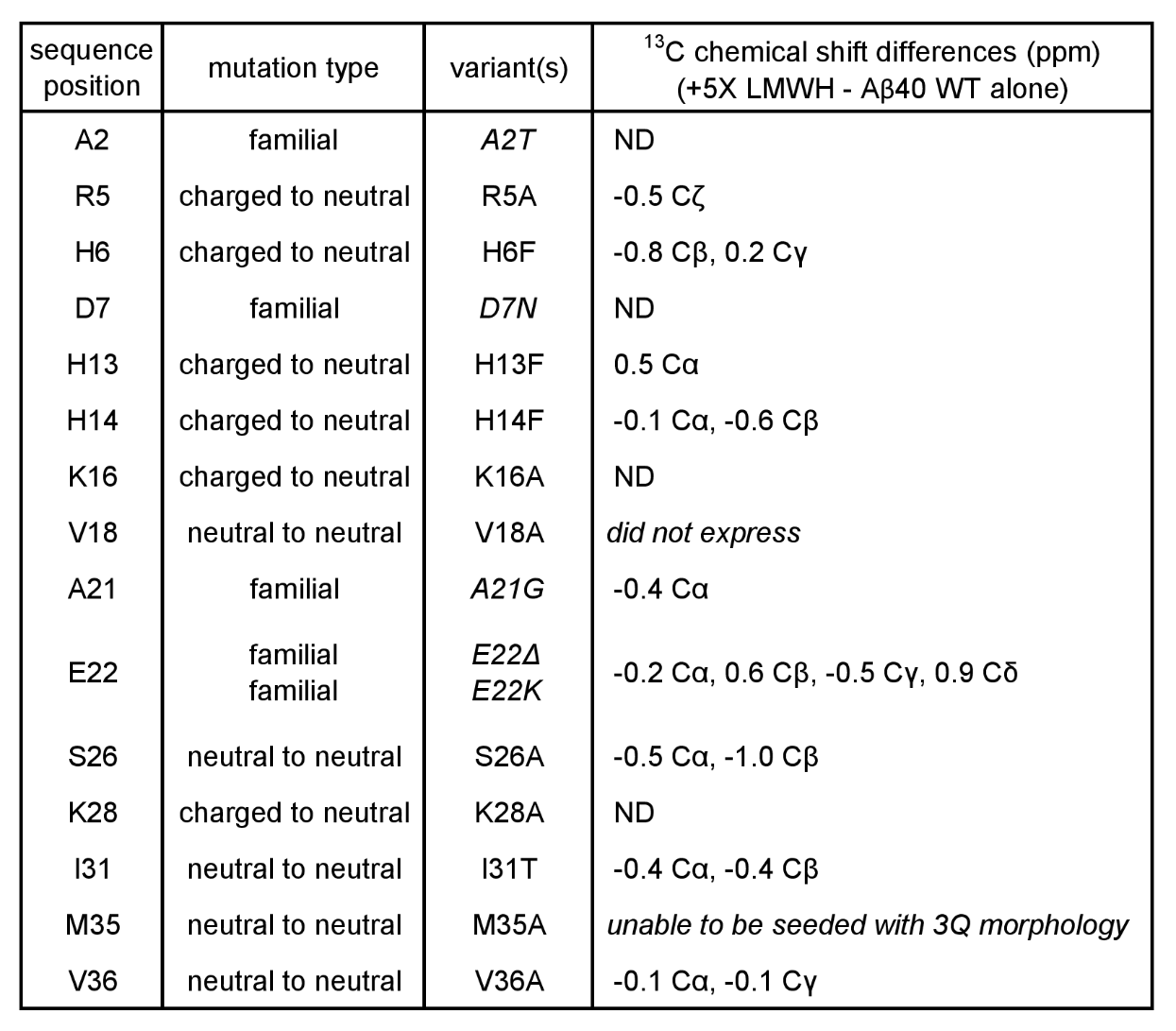


**Table S3**: Summary of Aβ40 variants characterized. Familial Alzheimer’s disease mutants are italicized in the third column. The right column shows chemical shift differences determined using ^13^C-^13^C DARR spectra of each 3Q fibril alone and in the presence of a five-fold molar excess of LMWH [8]. ND = not determined.

**Table S4**


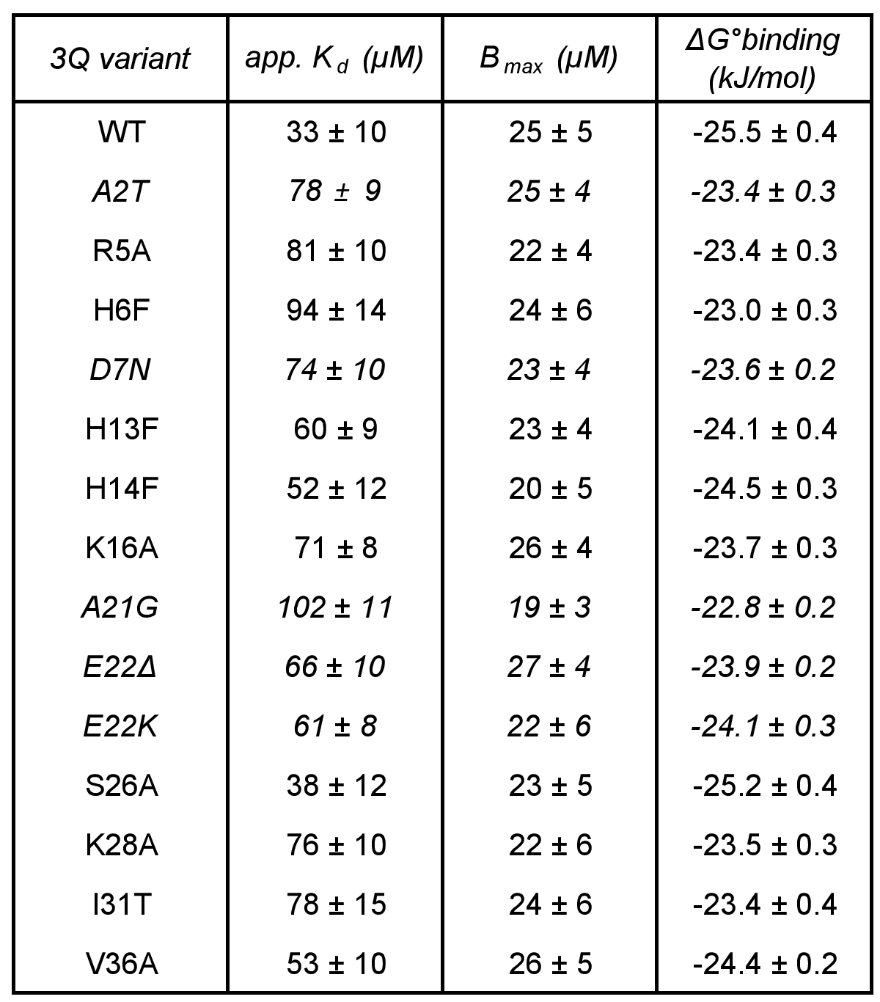


**Table S4**: Summary of binding energies of LMWH to Aβ40 3Q variants. K_d_, B_max_, and ΔG° binding values (with standard deviation) are described in the text and the raw data are shown in Figures 4 and S7. Variants in italics are single point substitution mutants implicated in familial Alzheimer’s disease.

**Table S5**

| PDB code | protein | heparin fragment^a^ | | resolution (Å) |
| --- | --- | --- | --- | --- |
| 4QFJ | Rat angiogenin | | Heparin disaccharide I-S (H1S) | 2.2 |
| 3OGX | Peptidoglycan recognition protein | | H1S | 2.8 |
| 3IN9 | Heparin lyase I | | H1S | 2.0 |
| 2NWG | CXCL12 | | H1S | 2.1 |
| 2FUT | Heparinase II | | H1S | 2.3 |
| 1U4L | Human RANTES | | H1S | 2.0 |
| 2HYU | Annexin A2 | | Heparin tetrasaccharide | 1.9 |
| 1SR5 | Anhydrothromin | | Heparin heptasaccharide (NT1) | 3.1 |
| 4R9W | Platelet factor 4 | | Trisulfoamino heparin pentasaccharide (NTO) | 2.5 |
| 4X7R | TarM mutant | | NTO | 2.2 |
| 3EVJ | Antithrombin | | NTO | 3.0 |
| 3KCG | Antithrombin-factor IXa | | Heparin pentasaccharide (NTP) | 1.7 |
| 5T03 | Heparan sulfate 6-O-sulfotransferase | | Heparin hexasaccharide | 2.1 |
| 4PXQ | D-glucuronyl C5-epimerase | | Heparin hexasaccharide | 2.2 |
| 3QMK | E2 domain of APLP1 | | Heparin hexasaccharide | 2.2 |
| 2HYV | Human annexin A2 | | Heparin hexasaccharide | 1.4 |

^a^Ligand PDB code given in brackets where available.

**Table S5**: Summary of the X-ray crystal structures of protein-heparin fragment complexes used in the computation analysis in Figure 6 of the main text.

**References**

[1] K.L. Stewart, E. Hughes, E.A. Yates, G.R. Akien, T.Y. Huang, M.A. Lima, et al. Atomic details of the interactions of glycosaminoglycans with amyloid-beta fibrils. J Am Chem Soc. 138 (2016) 8328-8331.

[2] A.K. Paravastu, R.D. Leapman, W.M. Yau, R. Tycko. Molecular structural basis for polymorphism in Alzheimer's beta-amyloid fibrils. Proc. Natl. Acad. Sci. U. S. A. 105 (2008) 18349-18354.

[3] M.T. Colvin, R. Silvers, B. Frohm, Y.C. Su, S. Linse, R.G. Griffin. High resolution structural characterization of A-beta(42) amyloid fibrils by magic angle spinning NMR. J. Am. Chem. Soc. 137 (2015) 7509-7518.

[4] A.T. Petkova, R.D. Leapman, Z.H. Guo, W.M. Yau, M.P. Mattson, R. Tycko. Self-propagating, molecular-level polymorphism in Alzheimer's beta-amyloid fibrils. Science. 307 (2005) 262-265.

[5] J.P. Colletier, A. Laganowsky, M. Landau, M.L. Zhao, A.B. Soriaga, L. Goldschmidt, et al. Molecular basis for amyloid-beta polymorphism. Proc. Natl. Acad. Sci. U. S. A. 108 (2011) 16938-16943.

[6] Y.L. Xiao, B.Y. Ma, D. McElheny, S. Parthasarathy, F. Long, M. Hoshi, et al. A beta(1-42) fibril structure illuminates self-recognition and replication of amyloid in Alzheimer's disease. Nat. Struct. Mol. Biol. 22 (2015) 499-505.

[7] D.S. Wishart, C.G. Bigam, J. Yao, F. Abildgaard, H.J. Dyson, E. Oldfield, et al. H-1, C-13 and N-15 chemical-shift referencing in biomolecular NMR. J. Biomol. NMR. 6 (1995) 135-140.

[8] J. Madine, M.J. Pandya, M.R. Hicks, A. Rodger, E.A. Yates, S.E. Radford, et al. Site-specific identification of an abeta fibril-heparin interaction site by using solid-state NMR spectroscopy. Angew. Chem. Int. Ed. 51 (2012) 13140-13143.
